# Arthroscopy-assisted Procedures in Hand and Wrist Surgery: An Update. Where Are We So Far?

**DOI:** 10.1055/s-0044-1779336

**Published:** 2024-06-22

**Authors:** Diego Figueira Falcochio, João Pedro Farina Brunelli, Ricardo Kaempf de Oliveira, Gustavo Mantovani Ruggiero

**Affiliations:** 1 Grupo de Cirurgia da Mão, Santa Casa de Misericórdia, São Paulo, SP, Brasil; 2 Serviço de Mão e Microcirurgia, Santa Casa de Misericórdia, Porto Alegre, RS, Brasil; 3 Handcenter, São Paulo, SP, Brasil

**Keywords:** arthroscopy, pseudarthrosis, scaphoid bone, synovial cyst, wrist

## Abstract

Wrist and hand arthroscopy, despite being an old tool, has gained popularity and advanced in assisting in the treatment of various injuries and conditions in the region in recent years. Dorsal, volar, ulnar, and radial accessory portals are used to reach all points of the carpal and hand joints. The minimal tissue damage, lesser injury to the capsule and its mechanoreceptors, the assessment of injuries associated with the reason for surgery, and aesthetically more favorable scars have attracted many doctors and their patients. As a result, there has been an increase in publications and diversifications of arthroscopic techniques. The aim of this update article is to present the advances and the evidence available in the literature to assist readers in their decision on which technique to use in the treatment of wrist and hand conditions.

## Introduction

Arthroscopy has become an indispensable tool in the arsenal of the contemporary hand surgeon. Most procedures can be performed arthroscopically or hybridly, allowing the use of smaller incisions and less damage to healthy tissues, a fundamental step towards early postoperative recovery and better functional results.

**Fig. 1:**
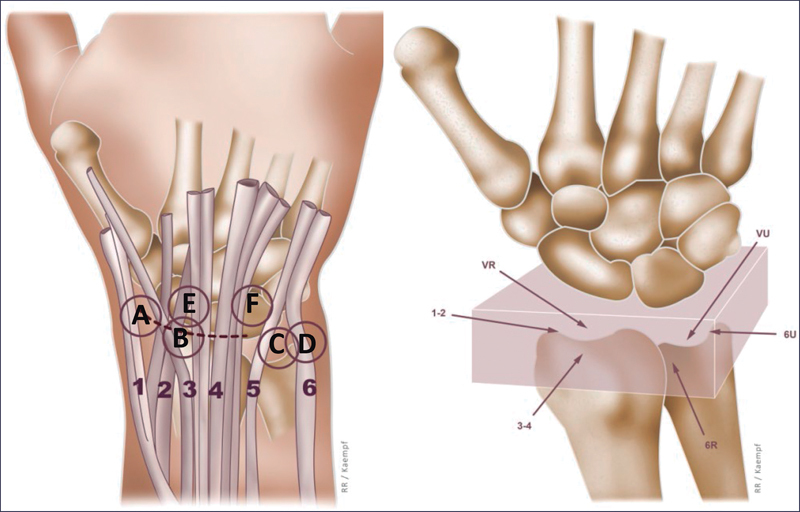
Wrist arthroscopy portals. A: portals 1-2, B: portals 3-4, C: portal 6R, D: portal 6U, E: radial mid-carpal portal (RMC), F: ulnar mid-carpal portal (UMC). VR: volar radial, VU: volar ulnar.

Through good anatomical knowledge and classic open procedures, the surgeon was able to introduce the arthroscope (about 3mm in diameter) into their daily routine, replacing larger incisions with small portals. The main portals used are the dorsal ones. In the radiocarpal joint the 3-4 (between the 3rd and 4th extensor compartments) and the 6R, radial to the extensor carpi ulnaris. In the midcarpal joint, the radial midcarpal (MCR) and ulnar midcarpal (UMC), both 1cm distal to the radiocarpals, radial and ulnar to the 4th extensor compartment. They were developed to cover most procedures, becoming routine and sufficient for the surgeon at the beginning of their learning curve. Assistance in joint reduction of fractures of the distal end of the radius and scaphoid and synovial cysts are great examples of this ( Fig. 1 ).

However, technical improvement and the emergence of more complex procedures have made it necessary to develop new techniques and new portals, with specific portals for each pathology, specific portals for certain joints and with greater exploration of volar portals. 1 2 3

The objective of this work is to point out how far we have come with arthroscopy to treat diseases and injuries that affect the wrists and hands.

To make reading easier, we will divide the article by the pathologies treated.

## Dorsal Synovial Cysts

The first technical description of arthroscopic resection of synovial cysts by Osterman and Raphael 4 demonstrated that dorsal cysts could be safely treated using this tool, generating similar recurrence rates and with lower rates of stiffness, postoperative pain and aesthetically unsatisfactory scarring. Later authors corroborated Osterman's results and described his variations of the technique. The most widespread are Kim's, 5 or radiocarpal technique, in which the cyst is approached through portals 3-4 and 4-5 (or 6R) and a capsulectomy is performed over the entire region of the cyst, and Mathoulin and Gras's, 6 or transcyst, in which the UMC and RMC portals are used. The shaver often passes through the cyst when creating the RMC portal, hence the name “transcyst”. The original technique includes reconstruction of the dorsal capsulo-scapholigamentary septum, better known by its acronym in DCSS. The authors of this article do not recommend this reconstruction method for cysts not associated with scapholunate ligament instability. In the case of instability, there are several options for repairing or reconstructing the ligament. It is worth noting that complete resection of the cyst is not necessary. The important thing is to create a “window” in the cyst wall to drain its contents.

The recurrence rate varies widely in the literature, suggesting the likelihood of bias in some studies, with the real incidence of lesion recurrence being unclear. A study pointed out some factors that may be associated with this, such as dominant side, female sex and young patients. 5 The location or not of the pedicle was not associated with an increase in recurrences, corroborating our clinical observation and that of other authors that this is not an obligatory step for the quality of the procedure. In fact, one study found that the vast majority of dorsal cysts present as a diffuse cystic mass and capsular redundancy, without the visualization of a classic pedicle image. 7 We also agree with this observation in our practice. Another important fact is that the presence of underlying pathologies does not seem to worsen postoperative results in terms of pain, function and cyst recurrence, as long as they are treated simultaneously in the same procedure. 8 This could be considered an advantage of treating cysts arthroscopically, in order to identify and treat these injuries, mostly injuries of the triangular fibrocartilage complex (TFCC) and injuries of the intrinsic ligaments of the first row. Some lesion associated with the dorsal synovial cyst is found in approximately 50% of patients undergoing arthroscopy for resection of the lesion. Most of these patients would remain undiagnosed if the resection was performed open. 8

As can be inferred, a significant portion of the studies places a great emphasis on the outcome of "recurrence" when discussing synovial cysts treated through arthroscopy. The use of methylene blue was introduced to facilitate the localization of the lesion and its pedicle. 9 There is no overwhelming evidence that its use is superior, requiring comparative work. In any case, the authors have good experience with both techniques, and the use of intraoperative dyeing is at the discretion of the surgeon.

## Volar Synovial Cysts

With the spread of arthroscopic treatment of dorsal cysts and the improvement of techniques, associated with the good functional and aesthetic results obtained, its use expanded to include volar cysts. Ho et al. 10 were the first to publish a series of arthroscopic treatment of volar cysts originating predominantly in the radiocarpal joint, showing good results. This represented a break with the paradigm that the presence of noble volar structures would prevent the procedure from being performed properly, with a greater risk of complications and recurrence. In fact, the procedure proved to be safer than the open technique in some studies, 11 except in cases of midcarpal joint cysts. Injury to noble structures in the open approach, especially the radial artery, tends to be more extensive. Other series have also demonstrated good satisfaction rates with arthroscopic treatment and a low rate of complications, such as recurrence. 12 13 A caveat presented in these series is the radiocarpal origin of the cyst, with mediocarpal cysts excluded from the casuistry. One justification provided by these authors is that, in addition to the greater instrumentation difficulty due to limited space, mediocarpal cysts are more superficial and farther from volar neurovascular structures, reducing the advantage of arthroscopic treatment and suggesting an open technique.

More recently, the same group that pioneered arthroscopic treatment of volar cysts published the technique and a small series of scaphotrapeziotrapezoid joint (STT) cysts, demonstrating that the method is feasible and safe with good results. 14 They used specific STT portals for lesion excision, further expanding the use of arthroscopy, now for mediocarpal cysts.

Another tool that has proven useful as an adjuvant in the treatment of cysts is ultrasound. 5 Its use is justified both by the greater ease in locating the lesion and by the protection of adjacent healthy structures, such as tendons, nerves and vessels. This same group, with the expansion of its series, observed that the aid of ultrasound is more useful for volar cysts than for dorsal cysts, with no recurrence of volar cysts in their series of more than 40 patients treated. 15

## Scaphotrapezoidal joint (STT)

Osteoarthritis of the STT joint (OA-STT) has a high incidence in the population, reaching more than 80% of individuals over 80 years of age in cadaveric studies. 16 Even in symptomatic cases, with pain at the base of the thumb and weakness of the pinch, making daily activities difficult, such as opening a bottle, other conditions usually coexist that justify the symptoms, such as trapeziometacarpal arthrosis and scapholunate injury/carpal instability. 17 Arthroscopy has become an important tool in the management of OA-STT as it allows, in the same procedure, the diagnosis and staging of the injury, the diagnosis of possible associated injuries and definitive treatment, which is difficult to achieve with open techniques, in which only the target joint will be addressed.

The treatment rationale should be guided by the presence of isolated OA-STT or concomitant carpometacarpal arthrosis (peritrapezial arthrosis) or carpal instability (DISI). 18 For cases of isolated OA-STT, some procedures can be performed with arthroscopic assistance, such as simple synovectomy, resection of the distal pole of the scaphoid, STT arthrodesis, capsular or tendon interposition arthroplasty and interposition arthroplasty with silicone implant or pyrocarbon. 19

STT is traditionally approached through the RMC portal for visualization, with the creation of the STT dorsal ulnar portal (STT-U) “from the outside in”, introducing an ulnar needle to the extensor pollicis longus (EPL), in line with the edge radial of the second metacarpal. More recently, new portals have been added, allowing access to all portions of the joint. Perez Carro et al. 20 introduced the radial dorsal portal of the STT (STT-R), made immediately radial to the abductor pollicis longus (APL) tendon. Baré et al. 21 described in the same year a palmar portal of the STT (STT-P) easily created by palpating the ALP, the scaphoid tubercle, the radial styloid and the base of the first metacarpal.

Regarding treatment, Ashwood et al. 22 demonstrated good short-term postoperative symptomatic relief with simple arthroscopic synovectomy, offering low morbidity in cases of isolated OA-STT, and can be a good tool before proceeding to more invasive treatments, similar to what has been performed on the knee for decades. Iida et al. 23 studied the impact of arthroscopic resection of the distal pole of the scaphoid and found good results, as long as the resection did not exceed 3 mm and there was no associated carpal instability (DISI). Luchetti et al. 24 found similar results, adding that the technique does not contraindicate cases of associated trapeziometacarpal arthrosis, as long as it is treated concomitantly. Mathoulin and Darin 25 had already published their series using this same technique, with the exception that, in the long term, an impingement phenomenon could occur at the joint level, justifying the addition of pyrocarbon implants to maintain physiological carpal height. This may also be advantageous in the case of concomitant DISI, where simple resection may worsen long-term instability, justifying some interposition. 18

STT arthroscopy can also be performed in combination with the trapeziometacarpal (TMC) joint approach, adding to the main procedure (trapeziectomy, hemitrapeziectomy with or without interposition) partial resection of the trapezoid and/or the distal pole of the scaphoid in cases of combined TMC-STT osteoarthritis, improving postoperative results in terms of mobility and pain. 18 19

STT arthrodesis, despite being widespread, does not appear to be a satisfactory solution, associated with a long period of immobilization, severe loss of range of motion and high rates of complications, such as non-union. 18

## Rhizarthrosis

The concepts of open procedures were brought into arthroscopy to offer options with greater preservation of the soft tissue envelope in trapeziometacarpal osteoarthritis. The most commonly used traditional techniques, despite showing good results in the literature, require significant capsuloligamentary violation and bone manipulation, with potential postoperative impacts in terms of pain and time to functional recovery. The lack of consistent evidence of superiority among traditional techniques (simple trapeziectomy, trapeziectomy with interposition, trapeziectomy with suspensionplasty) also leaves room for considering more tissue-preserving procedures. Arthroscopic hemitrapeziectomy is an example of this, allowing the resection of the contact surface between the metacarpal and the trapezium without violating the capsuloligamentary envelope. 26 The addition of interpositions, synthetic or biological material, such as tendons and fascia, has also been widely tested, being an alternative, however without consistent evidence of superiority to simple resection in controlled and randomized studies. 27 Studies with fat grafting as an alternative to interpositions have also been published, proclaiming a possible chondral regenerative effect induced by adipose stem cells. 28 More recently, techniques combining partial trapezius resection with button suspensionplasty have had the advantage of preventing metacarpal sinking in a proximal direction. 29 Arthrodesis, attractive to patients who require greater clamping force, also proved to be feasible through arthroscopy. 30 Even arthroscopic synovectomy without trapezius resection appears to offer good results in the short and medium term. 31 A meta-analysis demonstrated that arthroscopy-assisted techniques are a good alternative when conservative treatment fails, with less morbidity. 32

## Kienböck's disease

In order to determine the treatment for Kienböck Disease, in addition to the Litchmann classification, the Bain and Begg classification is used, 33 which is based on the number and location of “non-functional” joints of the lunate and its surroundings, diagnosed by arthroscopy. A nonfunctional joint is one that has extensive fibrillation, cracks, localized or extensive joint damage, and a floating joint surface. When diagnosing bad joints, partial wrist arthrodeses can be planned and openwork and fixation performed for these fusions.

## Fracture and Intra-articular Osteotomy of the Distal Radius Region

Arthroscopy is a tool to assist or check joint reduction in the distal region of the radius. Saab et al., 34 in their meta-analysis published in 2019, came to the conclusion that the use of arthroscopy helped in the diagnosis and simultaneous treatment of ligament injuries associated with fractures of the distal region of the radius, such as injury to the scapholunate ligament (SLL) or the triangular fibrocartilage complex (TFCC). However, there is no evidence in the literature that proves that arthroscopy guarantees a better reduction of joint fragments or better functional results.

In the authors' opinion, arthroscopy, for selected cases, brings many benefits, not only in the diagnosis of associated injuries, but also in the reduction of joint fragments and the certainty that the implants are outside the joint and that the fragments are stable enough for mobilization precocious.

To perform wrist arthroscopy in patients with fractures of the distal region of the radius, dry arthroscopy is recommended. 35 Not using the solution will avoid compartment syndrome, tissue infiltration and the presence of floating synovium, for example.

The recommended technique consists of performing metaphyseal reduction and positioning the locked plate and, only after this step, the wrist is placed under traction to perform the reduction or check the reduction of joint fractures and definitive fixation with screws. 36 For partial joint fractures, we can place the patient under traction and perform reduction and fixation with the aid of arthroscopy and fluoroscopy. Comminuted intra-articular fractures also benefit from arthroscopic assistance in reduction. 37 One should not forget to keep the optics in the 6R portal: over the head of the ulna, the camera does not interfere with the reduction and visualization of the dorsal fragments. 36

Viciously consolidated radius fractures bring significant harm to patients. Arthroscopy assists in assessing the viability of articular cartilage, determining locations for osteotomies, and allows for precise bone cuts. Similar to fractures, metaphyseal reduction should be performed without arthroscopy, positioning the plate, initiating the plan for articular osteotomies. Only after that, the patient is placed under traction, and the intra-articular osteotomy is completed. 38

## Scaphoid Fracture and Pseudoarthrosis

The role of arthroscopy in the treatment of scaphoid fractures and non-unions is progressively increasing. Slade et al. 39 published a series of 234 scaphoid fractures and non-unions treated with the aid of arthroscopy and fixed with an antegrade percutaneous method, with excellent consolidation results, reaching 99% in acute injuries, without the use of bone graft. Slutsky and Trevare 40 corroborated these results, pointing out the main objectives of arthroscopy: checking the quality of the reduction, checking the stability of the fixation with probe manipulation of the fragments and checking the position of the implants, confirming the absence of extrusion into the joint. It also pointed out its usefulness in the diagnosis of associated injuries, such as ligament injuries (transscaphoperilunar fracture-dislocation). These injuries were successfully treated arthroscopically in some series, and could replace open techniques, which until recently were the gold standard. 41 The possibility of grafting and approaching the ligament components by arthroscopy offers a more biological solution to the traditional open approach. Caloia et al. 42 found previously undiagnosed associated injuries in 15 of the 24 cases of scaphoid fractures treated arthroscopically, favoring the use of the tool. Geissler 43 also contributed to the topic, recommending percutaneous fixation associated with arthroscopy in athletes with scaphoid fractures as an alternative to conservative treatment, even in non-displaced fractures. Due to the prolonged period until consolidation, this method would favor the maintenance of function and an earlier return to sporting activities. In this article, the author also detailed his fixation method, which allows arthroscopic localization of the screw entry point, without the need for multiple fluoroscopy and without the need for hyperflexion of the wrist.

**Fig. 2:**
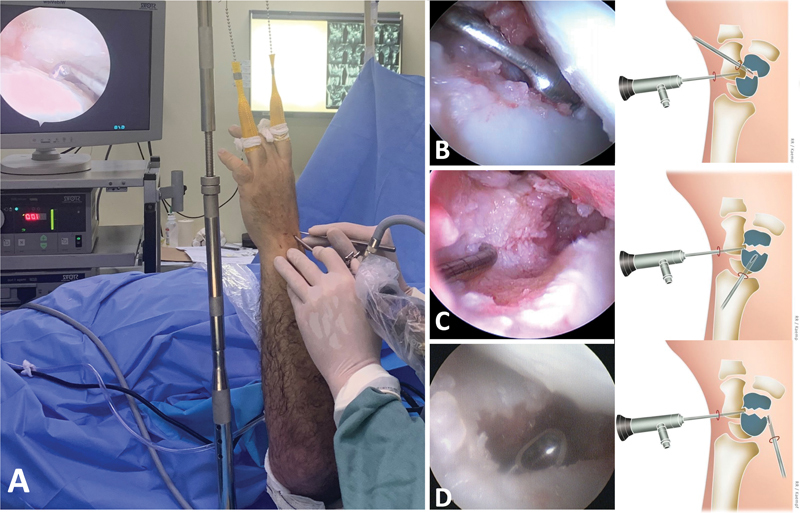
Wrist arthroscopy for scaphoid non-union treatment. A: patient positioning. B: Scrapping of the scaphoid non-union with a curette through the radial mid-carpal portal. C: Scrapping and positioning through the radiocarpal portal. D: Scrapping through the volar aspect of the scaphoid.

The good results in acute scaphoid fractures and fracture-dislocations have led to many studies on non-unions. Several authors have already demonstrated that arthroscopic treatment of these injuries is not only feasible, but also presents similar or superior results to traditional techniques, when well indicated. Cognet et al. 44 published a series of 23 treated patients, with a consolidation rate of 100%. They highlighted that the arthroscopic view facilitates the removal of all devitalized tissue and that positioning in traction helps to reestablish the normal realignment of the bone ( Fig. 2 ). Ecker et al. 45 exposed the good results even for cases of pseudoarthrosis of the proximal pole, with high union rates. Initially, pseudarthroses with large humpback deformity and great instability were excluded from arthroscopic treatment series, however it is known that even these injuries can be treated with the aid of arthroscopy, with good results. Kim et al. 46 emphasize that, even though there is limited potential for restoring the normal length of the scaphoid in unstable pseudarthrosis, the functional results are satisfactory, possibly due to less aggression to healthy tissues and restricted vascular violation in relation to open techniques. The same applies to the early stages of SNAC, which currently does not represent a contraindication. Regarding the possibility of dispensing with the use of a graft, studies indicate that, in cases of stable pseudarthrosis and without carpal collapse / DISI, fixation without the use of a graft is sufficient to achieve union. 47

The scope of arthroscopy in the treatment of acute and chronic scaphoid injuries has been shown to be broad, with advantages over traditional techniques in terms of allowing the identification and treatment of associated pathologies, such as intrinsic ligament injuries, in allowing the optimal positioning of implants in the percutaneous fixations, aiding in debridement and reduction of fragments under direct visualization and greater preservation of the soft tissue envelope, favoring consolidation and good functional results.

## Scapholunate Ligament (SLL) Injury

The treatment of scapholunate ligament injuries is one of the greatest challenges in hand surgery. Many of these injuries go unnoticed in the acute phase, when repair is possible. Lack of adequate treatment leads to a pattern of carpal osteoarthritis known as scapholunate advanced collapse (SLAC). 48

In order to choose the best treatment for the patient, Garcia-Elias et al. 49 formulated 5 questions regarding important parameters for these injuries and described a 6-stage classification for them.

There are a myriad of possible treatments, which aim to restore the normal relationship between the scaphoid and lunate, recovering mobility, strength and function of the wrist in each of these stages. None proved to be absolutely superior to the others.

In this context, arthroscopy-assisted techniques for repair, reconstruction or salvage techniques have emerged and are emerging, with the idea of being less invasive, causing less morbidity and providing better results than conventional techniques.

For stage I, when the dorsal LES is intact, also known as pre-dynamic injury, percutaneous or arthroscopy-assisted fixation is indicated, with or without ligamentoplasty and dorsal capsulodesis. 50

For stage II, when there is rupture of the dorsal and volar SLL, ligamentoplasty and dorsal 50 e and volar 36 capsulodesis, can be performed, with scapholunate fixation with Kirschner wires or scapholunate screw (RASL) assisted by arthroscopy.

In stages III (complete, irreparable SLL injury) and IV (stage III with reducible scaphoid rotation), different types of reconstruction of the scapholunate ligament can be performed. The classic techniques are dorsal capsulodesis or Brunelli reconstruction or Brunelli modified by Garcia-Elias. The techniques that are currently gaining more followers are those that perform 360 ^o^ repair, such as that of Corella et al. 51

For stages V (irreducible scaphoid rotation) and VI (arthrosis secondary to SLL injury), partial and total arthrodesis of the wrist and resection of the proximal row of the carpal bones are indicated. There are several authors in the literature who demonstrate that it is feasible to perform these procedures assisted by arthroscopy. There is still no evidence, however, that minimally invasive procedures are advantageous over traditional techniques.

## Perilunar Carpal Dislocation and Perilunar Carpal Fracture-Dislocation

Since wrist arthroscopy can assist in the reduction and fixation of radius and scaphoid fractures, as well as in the repair of carpal ligaments, it has become an option for aiding in the reduction and treatment of carpal fracture-dislocations. Although there is a study in the literature demonstrating the superiority of arthroscopic assistance, 52 we will need more articles to ensure that this is the best technique. However, we advise caution in indicating arthroscopy for these injuries, as extensive capsular damage and significant instability pose significant technical challenges in the arthroscopic procedure.

## Injury of the Triangular Fibrocartilage Complex (TFCC)

This is one of the most indisputable indications for wrist arthroscopy today. Since Palmer's classic article, 53 which classified traumatic and degenerative TFCC injuries, much has been studied about the anatomy and functions of this ligament complex. Those who have experienced open surgery for ulnar disinsertion of the TFCC have often had difficulty both in identifying the correct tissue and in defining the site for reinsertion of the TFCC.

Performing TFCC debridement and evaluating chondropathy of the lunate, triquetrum and head of the ulna via an open approach seems a distant reality. The tissue damage would be very large and the assessment incomplete.

**Fig. 3:**
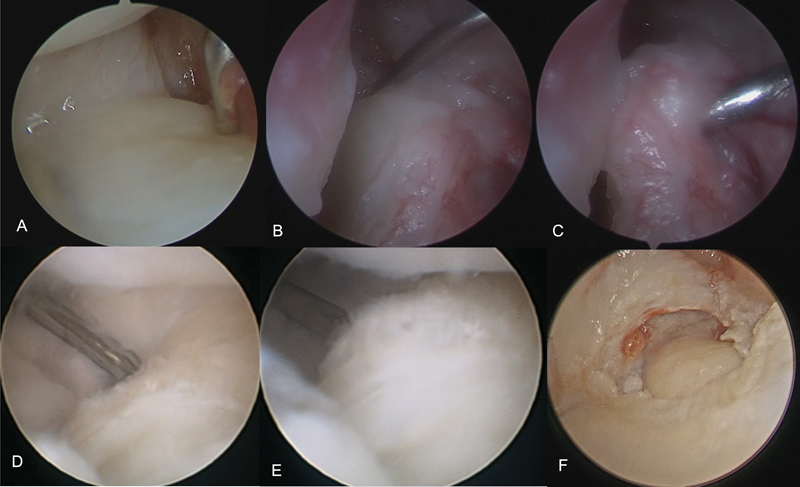
Types of triangular fibrocartilage complex (TFCC) injuries. A: Distal detachment of the TFCC, class 1. B: Proximal and distal detachment of the TFCC, class 2. C: Positive "hook" test for the injury shown in figure B (class 2). D: TFCC class 4 injury with friable edges. E: The same patient as shown in figure B, with significant instability of the remaining TFCC. F: Massive irreparable TFCC class 4 injury.

The 2008 article by Anderson et al. 54 demonstrated no significant differences in patient function and mobility between arthroscopic and open TFCC repair. However, only the latter performed foveal reinsertion of the ligament. In 2009, Atzei 1 published his work on arthroscopic foveal reinsertion of the TFCC, which gained popularity and gained recognition around the world. In this same article, he proposed a TFCC classification for ulnar peripheral traumatic injuries (Palmer Class 1B) ( Fig. 3 ).

This classification allowed an algorithm to be designed for the treatment of peripheral ulnar TFCC injuries and their sequelae. Injuries without foveal disinsertion can be repaired with peripheral stitches between the capsule and the TFCC, injuries with proximal avulsion must be reinserted into the fovea, irreparable injuries must be reconstructed and arthroplasties and arthrodeses (Sauvè-Kapandji) can be performed for patients with injuries cartilaginous.

Since 2009, several other authors have launched techniques for foveal TFCC reinsertion via arthroscopic 55 56 and open methods. 57

Arthroscopic techniques for TFCC reconstruction with tendon grafts have begun to emerge. The open technique of Adams and Berger 58 is the gold standard for reconstruction of the ligament complex. However, there are several techniques gaining popularity for arthroscopic reconstruction. 59 60 61 We believe that, over time, anatomical techniques with less joint and mechanoreceptor damage will replace open techniques.

Obviously, the distal radio-ulnar joint must be observed in all its complexity and not just the TFCC to make decisions regarding the treatment to be implemented. 62

## Discussion

Arthroscopy in the wrist and hand joints has developed very rapidly in recent years. The European Wrist Arthroscopy Society has greatly contributed to this leap in knowledge and practice, as it brought together different schools in Europe and the world around the topic.

For several procedures, we believe we are better off with the help of arthroscopy than without it. However, we must remember that arthroscopy is a method, a tool to get where we want and not the purpose of the treatment.

## Conclusion

Wrist and hand arthroscopy provides assistance in the diagnosis and treatment of the vast majority of injuries and conditions that affect the region.
