# Supplementary material for: Arthroscopy-assisted Procedures in Hand and Wrist Surgery: An Update. Where Are We So Far?
Source: Rev Bras Ortop (Sao Paulo). 2024 Jun 22;59(3):e349–57. doi: 10.1055/s-0044-1779336 (PMC11193588; doi:10.1055/s-0044-1779336)
Supplement: Related texts

# Research article: Procedimentos assistidos por artroscopia no punho e na mão: Atualização. Onde nós estamos?

**Keywords:** artroscopia, cisto sinovial, osso escafoide, pseudartrose, punho

## Abstract

A artroscopia de punho e mão, apesar de ser uma ferramenta antiga, tem ganhado popularidade e avançado no auxílio do tratamento das mais diversas lesões e doenças da região nos últimos anos. Utilizam-se portais acessórios dorsais, volares, ulnares e radiais para se alcançar todos os pontos das articulações do carpo e da mão. O menor dano tecidual, a menor lesão da cápsula e de seus mecanoceptores, a avaliação de lesões associadas ao motivo da cirurgia e as cicatrizes esteticamente mais favoráveis têm atraído muitos médicos e seus pacientes. Com isso, houve um aumento das publicações e diversificações de técnicas artroscópicas. O objetivo deste artigo de atualização é mostrar os avanços e o que temos de evidência na literatura para apoiar os leitores na sua decisão sobre qual técnica utilizar nos tratamentos das doenças do punho e da mão.

## Introdução

A artroscopia tornou-se uma ferramenta indispensável no arsenal do cirurgião da mão contemporâneo. Grande parte dos procedimentos podem ser realizados de forma artroscópica ou híbrida, permitindo o emprego de incisões menores e menos dano aos tecidos sadios, passo fundamental para a recuperação pós-operatória precoce e melhores resultados funcionais.

Através de um bom conhecimento anatômico e de procedimentos clássicos abertos, o cirurgião pôde introduzir o artroscópio (de cerca de 3mm de diâmetro) na sua rotina diária, substituindo incisões maiores por pequenos portais. Os principais portais utilizados são os dorsais. Na articulação radiocarpal o 3-4 (entre o 3 ^o^ e 4 ^o^ compartimentos extensores) e o 6R, radial ao extensor ulnar do carpo. Na articulação mediocarpal o MCR (mediocarpal radial) e MCU (mediocarpal ulnar), ambos 1cm distal aos radiocarpais, radial e ulnar ao 4° compartimento extensor. Eles foram desenvolvidos para atender à maioria dos procedimentos, tornando-se rotineiros e suficientes para o cirurgião no início da sua curva de aprendizado. O auxílio na redução articular das fraturas da extremidade distal do rádio e do escafoide e os cistos sinoviais são ótimos exemplos disso ( Fig. 1 ).

**Fig. 1:** Portais para artroscopia do punho. A: portal 1-2, B: portal 3-4, C: portal 6R, D: portal 6U, E: portal médio-carpal radial (MCR), F: portal médio-carpal ulnar (MCU).VR: volar radial, VU: volar ulnar.

Entretanto, o aprimoramento técnico e o surgimento de procedimentos mais complexos fizeram necessário o desenvolvimento de novas técnicas e novos portais, com portais específicos para cada patologia, portais específicos para determinadas articulações e com maior exploração dos portais volares. 1 2 3

O objetivo deste trabalho é apontar até onde chegamos com a artroscopia para tratamento das doenças e lesões que acometem os punhos e mãos.

Para facilitar a leitura, vamos dividir o artigo pelas patologias tratadas.

## Cistos Sinoviais Dorsais

A primeira descrição técnica de ressecção artroscópica dos cistos sinoviais por Osterman e Raphael 4 demonstrou que os cistos dorsais podiam ser tratados com segurança através dessa ferramenta, gerando índices de recidiva semelhantes e com menores taxas de rigidez, dor pós-operatória e cicatriz esteticamente insatisfatória. Autores posteriores corroboraram os resultados de Osterman e descreveram suas variações da técnica. As mais difundidas são a de Kim, 5 ou técnica radiocarpal, em que o cisto é abordado através dos postais 3-4 e 4-5 (ou 6R) e uma capsulectomia é realizada em toda a região do cisto, e a de Mathoulin e Gras, 6 ou transcisto, em que os portais MCU e MCR são utilizados. O *shaver* com frequência atravessa o cisto na confecção do portal MCR, por isso o nome “transcisto”. A técnica original inclui a reconstrução do septo capsuligamentar dorsal, mais conhecido pela sua sigla em inglês (DCSS). Os autores deste artigo não recomendam esse método de reconstrução para cistos não associados a instabilidade do ligamento escafolunar. No caso de instabilidade, há diversas opções para reparo ou reconstrução do ligamento. Vale ressaltar que não é necessária a ressecção completa do cisto. O importante é realizar uma “janela” da parede do cisto para a drenagem de seu conteúdo.

A taxa de recidiva varia amplamente na literatura, sugerindo a probabilidade de vieses em alguns estudos, não estando clara a real incidência de recorrência da lesão. Um estudo apontou alguns fatores que podem estar associados a isso, como lado dominante, sexo feminino e pacientes jovens. 5 A localização ou não do pedículo não se mostrou associada ao aumento das recidivas, corroborando nossa observação clínica e a de outros autores de que isso não é passo obrigatório para a qualidade do procedimento. De fato, um estudo apontou que a grande maioria dos cistos dorsais se apresenta como uma massa difusa cística e uma redundância capsular, sem a visualização de uma imagem pedicular clássica. 7 Concordamos também com essa observação na nossa prática. Outro dado importante é que a presença de patologias subjacentes parece não piorar os resultados pós-operatórios em relação a dor, função e recorrência do cisto, contanto que sejam tratadas simultaneamente no mesmo procedimento. 8 Isso poderia ser considerado uma vantagem de tratar os cistos artroscopicamente, a fim de identificar e tratar essas lesões, em sua maioria lesões do complexo da fibrocartilagem triangular (CFCT) e lesões dos ligamentos intrínsecos da primeira fileira. Encontra-se alguma lesão associada ao cisto sinovial dorsal em cerca de 50% dos pacientes submetidos à artroscopia para ressecção da lesão. A imensa parte destes pacientes ficaria sem o diagnóstico, caso a ressecção fosse feita aberta. 8

Como pode ser depreendido, grande parte dos trabalhos valorizam muito o desfecho “recidiva” quando falam dos cistos sinoviais tratados por artroscopia. O uso de azul de metileno foi trazido para facilitar a localização da lesão e do pedículo. 9 Não há evidência contundente que seu uso é superior, carecendo de trabalhos comparativos. De qualquer modo, os autores têm boa experiência com as duas técnicas, ficando a critério do cirurgião o emprego do tingimento intraoperatório.

## Cistos Sinoviais Volares

Com a difusão do tratamento artroscópico dos cistos dorsais e o aprimoramento das técnicas, associados aos bons resultados funcionais e estéticos obtidos, o uso expandiu-se para os cistos volares. Ho et al. 10 foram os primeiros a publicar uma série de tratamento artroscópico dos cistos volares originados predominantemente na articulação radiocarpal, apresentando bons resultados. Isso representou a quebra do paradigma que a presença das estruturas nobres volares impediria que o procedimento fosse realizado de maneira adequada, com maior risco de complicações e recidiva. Na verdade, o procedimento mostrou-se mais seguro que a técnica aberta em alguns trabalhos, 11 exceto nos casos de cistos da articulação mediocarpal. A lesão das estruturas nobres na abordagem aberta, especialmente a artéria radial, tende a ser mais extensa. Outras séries também demonstraram bons índices de satisfação com o tratamento artroscópico e baixo índice de complicações, como recidiva. 12 13 Uma ressalva apresentada nessas séries é a origem radiocarpal do cisto, excluindo-se da casuística cistos mediocarpais. Uma justificativa desses autores é que, além da maior dificuldade na instrumentação, devido ao menor espaço, os cistos mediocarpais seriam mais superficiais e distantes das estruturas neurovasculares volares, diminuindo a vantagem do tratamento artroscópico e sugerindo a técnica aberta.

Mais recentemente, o mesmo grupo que foi pioneiro no tratamento artroscópico dos cistos volares publicou a técnica e uma pequena série de cistos da articulação escafotrapeziotrapezoide (STT), demonstrando que o método é factível e seguro, com bons resultados. 14 Eles utilizaram portais específicos da STT na ressecção das lesões, expandindo ainda mais o uso da artroscopia, agora para cistos mediocarpais.

Outra ferramenta que se mostrou útil como adjuvante no tratamento dos cistos é a ultrassonografia. 5 Seu uso é justificado tanto pela maior facilidade na localização da lesão quanto pela proteção das estruturas sadias adjacentes, como tendões, nervos e vasos. Esse mesmo grupo, com a expansão de sua casuística, observou que o auxílio do ultrassom é mais útil para os cistos volares que para os dorsais, não apresentando nenhuma recidiva de cisto volar na sua série de mais de 40 pacientes tratados. 15

## Articulação Escafotrapeziotrapezoidal (STT).

A osteoartrite da articulação STT (OA-STT) tem alta incidência na população, chegando a mais de 80% dos indivíduos com idade superior a 80 anos em estudos cadavéricos. 16 Mesmo nos casos sintomáticos, com dor na base do polegar e fraqueza de pinça, dificultando atividades do dia-a-dia, como abrir uma garrafa, habitualmente coexistem outras condições que justificam os sintomas, como artrose trapeziometacarpal e a lesão escafolunar/instabilidade carpal. 17 A artroscopia tornou-se uma ferramenta importante no manejo da OA-STT por permitir que, em um mesmo procedimento, possa realizar-se o diagnóstico e o estadiamento da lesão, o diagnóstico de possíveis lesões associadas e o tratamento definitivo, dificilmente possível com técnicas abertas, em que apenas a articulação alvo será abordada.

O racional de tratamento deve ser guiado pela presença de OA-STT isolada ou pela concomitância de artrose carpometacarpal (artrose peritrapezial) ou instabilidade carpal (DISI). 18 Para casos de OA-STT isolada, alguns procedimentos podem ser realizados com auxílio artroscópico, como a sinovectomia simples, a ressecção do polo distal do escafoide, a artrodese STT, a artroplastia de interposição capsular ou tendínea e a artroplastia de interposição com implante de silicone ou pirocarbono. 19

A STT é abordada tradicionalmente pelo portal MCR para visualização, com a confecção do portal dorsal ulnar da STT (STT-U) “de fora para dentro”, introduzindo uma agulha ulnar ao extensor longo do polegar (EPL), em linha com a borda radial do segundo metacarpal. Mais recentemente, novos portais foram adicionados, permitindo o acesso a todas as porções da articulação. Perez Carro et al. 20 introduziram o portal dorsal radial da STT (STT-R), confeccionado imediatamente radial ao tendão do abdutor longo do polegar (APL). Baré et al. 21 descreveram no mesmo ano um portal palmar da STT (STT-P) facilmente confeccionado palpando-se o ALP, o tubérculo do escafoide, o estiloide radial e a base do primeiro metacarpal.

Em relação ao tratamento, Ashwood et al. 22 demonstraram bom alívio sintomático pós-operatório a curto prazo com a sinovectomia simples artroscópica, oferecendo baixa morbidade em casos de OA-STT isolada, podendo ser uma boa ferramenta antes de proceder a tratamentos mais invasivos, à semelhança do que é realizado no joelho há décadas. Iida et al. 23 estudaram o impacto da ressecção artroscópica do polo distal do escafoide e encontrou bons resultados, desde que a ressecção não excedesse os 3mm e que não houvesse instabilidade carpal associada (DISI). Luchetti et al. 24 encontraram resultados semelhantes, acrescentando que não contraindica a técnica para casos de artrose trapeziometacarpal associada, desde que tratada concomitantemente. Mathoulin e Darin 25 já haviam publicado sua série com essa mesma técnica, com a ressalva que, a longo prazo, poderia ocorrer um fenômeno de *impingement* a nível da articulação, justificando o acréscimo de implantes de pirocarbono para manter a altura carpal fisiológica. Isso também pode ser vantajoso no caso de DISI concomitante, em que a simples ressecção pode piorar a instabilidade a longo prazo, justificando alguma interposição. 18

A artroscopia da STT também pode ser realizada em combinação com a abordagem da articulação trapeziometacarpal (TMC), acrescentando-se ao procedimento principal (trapeziectomia, hemitrapeziectomia com ou sem interposição) a ressecção parcial do trapezoide e/ou do polo distal do escafoide nos casos de osteoartrite combinada TMC-STT, melhorando os resultados pós-operatórios em termos de mobilidade e dor. 18 19

A artrodese STT, apesar de amplamente difundida, não parece ser uma solução satisfatória, associada a longo período de imobilização, grave perda da amplitude de movimento e altas taxas de complicações, como a não-consolidação. 18

## Rizartrose

Os conceitos dos procedimentos abertos foram trazidos para a artroscopia para oferecer opções com maior preservação do envelope de partes moles na artrose trazpeziometacarpal. As técnicas tradicionais mais utilizadas, apesar de demonstrarem bons resultados na literatura, exigem grande violação capsuloligamentar e manipulação óssea, com possível impacto no pós-operatório em relação à dor e ao tempo até a recuperação funcional. O fato de não haver evidências consistentes de superioridade entre as técnicas tradicionais em termos de desfecho (trapeziectomia simples, trapeziectomia com interposição, trapeziectomia com suspensoplastia) também abre espaço para se pensar em procedimentos mais preservadores. A hemitrapeziectomia artroscópica é um exemplo disso, permitindo a ressecção da superfície de contato entre o metacarpal e o trapézio sem violação do envelope capsuloligamentar. 26 O acréscimo de interposições, sintéticas ou de material biológico, como tendões e fáscias, também foi amplamente testado, sendo uma alternativa, no entanto sem evidências consistentes de superioridade à ressecção simples em trabalhos controlados e randomizados. 27 Estudos com enxerto de gordura como alternativa às interposições também foram publicados, pregando um possível efeito regenerativo condral induzido por células-tronco adiposas. 28 Mais recentemente, técnicas combinando a ressecção parcial do trapézio com suspensoplastia com botões trouxeram a vantagem de prevenir o afundamento do metacarpal em direção proximal. 29 A artrodese, atrativa a pacientes que necessitam maior força de pinça, também se mostrou factível por artroscopia. 30 Até mesmo a sinovectomia artroscópica sem ressecção do trapézio parece oferecer bons resultados a curto e médio prazo. 31 Uma meta-análise demonstrou que técnicas assistidas por artroscopia se mostram como boa alternativa na falha do tratamento conservador, com menos morbidade. 32

## Doença de Kienböck

A fim de ditar o tratamento para a Doença de Kienböck, além da classificação de Litchmann, utiliza-se a classificação de Bain e Begg, 33 que se baseia no número e localização de articulações “não funcionais” do semilunar e ao seu redor, diagnosticadas por artroscopia. Uma articulação não funcional é aquela que tem fibrilação extensa, fissuras, lesão articular localizada ou extensa e superfície articular flutuante. Ao se diagnosticar articulações ruins, pode-se se planejar as artrodeses parciais do punho e realizar a cruentização e fixação para essas fusões.

## Fratura e Osteotomia Intra-articular da Região Distal do Rádio

A artroscopia é uma ferramenta para o auxílio ou conferência da redução articular da região distal do rádio. Saab et al., 34 em sua metanálise publicada em 2019, chegou à conclusão de que o uso da artroscopia auxiliou no diagnóstico e tratamento simultâneo de lesões ligamentares associadas às fraturas da região distal do rádio, como lesão do ligamento escafolunar (LES) ou do complexo da fibrocartilagem triangular (CFCT). Contudo, não há evidência na literatura que comprove que a artroscopia garanta uma melhor redução dos fragmentos articulares ou resultados funcionais melhores.

Na opinião dos autores, a artroscopia, para casos selecionados, traz muitos benefícios, não só no diagnóstico de lesões associadas, como na redução dos fragmentos articulares e na certeza de que os implantes estão fora da articulação e que os fragmentos estão suficientemente estáveis para mobilização precoce.

Para realizar a artroscopia do punho em pacientes com fraturas da região distal do rádio, aconselha-se a artrosocopia seca. 35 Não utilizar soro irá evitar síndrome compartimental, infiltração de tecidos e presença de sinóvia flutuante, por exemplo.

A técnica preconizada consiste em realizar redução metafisária e posicionamento da placa bloqueada e, apenas após esta etapa, coloca-se o punho sob tração para realizar a redução ou conferir a redução das fraturas articulares e fixação definitiva com os parafusos. 36 Para fraturas articulares parciais, podemos colocar o paciente sob tração e realizar a redução e fixação com auxílio da artroscopia e fluoroscopia. As fraturas intra-articulares cominutas também se beneficiam do auxílio artroscópico na redução. 37 Não se deve esquecer de manter a óptica no portal 6R: sobre a cabeça da ulna a câmera não atrapalha a redução e visualização dos fragmentos dorsais. 36

As fraturas viciosamente consolidadas do rádio trazem grande prejuízo para os pacientes. A artroscopia auxilia a verificar a viabilidade da cartilagem articular, os locais para as osteotomias e possibilita a confecção dos cortes ósseos no ponto exato. Da mesma forma que nas fraturas, a redução metafisária deve ser feita sem a escopia, posiciona-se a placa, inicia-se o plano das osteotomias articulares e, apenas após, coloca-se o paciente sob tração e se completa a osteotomia por via intra-articular. 38

## Fratura e Pseudoartrose do Escafoide

O papel da artroscopia no tratamento das fraturas e pseudartroses do escafoide aumenta progressivamente. Slade et al. 39 publicaram uma série de 234 fraturas e pseudoartroses do escafoide tratadas com auxílio de artroscopia e fixadas com método percutâneo anterógrado, com excelentes resultados de consolidação, chegando a 99% nas lesões agudas, sem uso de enxerto ósseo. Slutsky e Trevare 40 corroboraram esses resultados, apontando os principais objetivos da artroscopia: verificar a qualidade da redução, verificar a estabilidade da fixação com a manipulação com *probe* dos fragmentos e verificar a posição dos implantes, confirmando a ausência de extrusão para a articulação. Também apontou a utilidade no diagnóstico de lesões associadas, como lesões ligamentares (fratura-luxação transescafoperilunar). Essas lesões foram tratadas com sucesso artroscopicamente em algumas séries, podendo substituir as técnicas abertas, até pouco tempo padrão-ouro. 41 A possibilidade de enxertia e abordagem dos componentes ligamentares por artroscopia oferece uma solução mais biológica à tradicional abordagem aberta. Caloia et al. 42 encontraram lesões associadas não diagnosticadas previamente em 15 dos 24 casos de fraturas do escafoide tratadas artroscopicamente, favorecendo o uso da ferramenta. Geissler 43 também contribuiu com o tema, recomendando a fixação percutânea associada à artroscopia em atletas com fraturas do escafoide como alternativa ao tratamento conservador, mesmo nas fraturas não desviadas. Devido ao prolongado período até consolidação, esse método favoreceria a manutenção da função e o retorno mais precoce às atividades esportivas. Neste artigo, o autor também detalhou seu método de fixação, que permite a localização artroscópica do ponto de entrada do parafuso, sem necessidade de múltiplas fluoroscopias e sem necessidade de hiperflexão do punho.

Os bons resultados nas fraturas e fraturas-luxações agudas do escafoide suscitaram muitos estudos para as pseudartroses. Diversos autores já demonstraram que o tratamento artroscópico dessas lesões não só é factível, como apresenta resultados semelhantes ou superiores às técnicas tradicionais, quando bem indicados. Cognet et al. 44 publicaram uma série de 23 pacientes tratados, com taxa de consolidação de 100%. Ressaltou que a visão artroscópica facilita a remoção de todo o tecido desvitalizado e que o posicionamento na tração auxilia no restabelecimento do realinhamento normal do osso ( Fig. 2 ). Ecker et al. 45 expôs os bons resultados mesmo para casos de pseudoartrose do polo próximal, com altas taxas de união. Inicialmente, as pseudoartroses com grande deformidade em *humpback* e grande instabilidade eram excluídas das séries de tratamento artroscópico, no entanto se sabe que mesmo essas lesões podem ser tratadas com auxílio de artroscopia, com bons resultados. Kim et al. 46 ressaltam que, mesmo havendo potencial limitado para restauração do comprimento normal do escafoide nas pseudoartroses instáveis, os resultados funcionais são satisfatórios, possivelmente pela menor agressão aos tecidos sadios e violação vascular restrita em relação às técnicas abertas. O mesmo se aplica aos estágios iniciais do SNAC, que atualmente não representa contraindicação. Em relação à possibilidade de dispensa do uso de enxerto, estudos apontam que, em caso de pseudartroses estáveis e sem colapso carpal / DISI, a fixação sem emprego de enxerto é suficiente para alcançar a união. 47

**Fig. 2:** Artroscopia do punho para o tratamento da pseudartrose do escafoide. A: posicionamento do paciente. B: Cruentização da pseudartrose com cureta por portal médio-cárpico radial. C: Cruentização e posicionamento com portal rádio-cárpico. D: Cruentização por volar do escafoide.

O alcance da artroscopia no tratamento das lesões agudas e crônicas do escafoide tem se mostrado amplo, com vantagens em relação às técnicas tradicionais no sentido de permitir a identificação e tratamento de patologias associadas, como lesões ligamentares intrínsecas, em permitir o posicionamento ótimo dos implantes nas fixações percutâneas, no auxílio no desbridamento e na redução dos fragmentos sob visualização direta e na maior preservação do envelope de partes moles, favorecendo a consolidação e bons resultados funcionais.

## Lesão do Ligamento Escafolunar (LES)

O tratamento da lesão do ligamento escafolunar é um dos grandes desafios em cirurgia da mão. Muitas destas lesões passam desapercebidas na fase aguda, quando o reparo é possível. A falta de tratamento adequado leva a um padrão de artrose do carpo conhecido como colapso avançado secundário à lesão do ligamento escafolunar (sigla em inglês SLAC). 48

A fim de se escolher o melhor tratamento para o paciente, Garcia-Elias et al. 49 formulou 5 perguntas de parâmetros importantes para estas lesões e descreveu uma classificação de 6 estágios para elas.

Há uma miríade de tratamentos possíveis, que visam resgatar a relação normal entre o escafoide e semilunar, recuperar a mobilidade, a força e a função do punho em cada um destes estágios. Nenhuma se mostrou absolutamente superior às demais.

Nesse âmbito, surgiram e surgem técnicas assistidas por artroscopia para reparo, reconstrução ou técnicas de salvação, com a ideia de serem menos invasivas, causarem menor morbidade e proporcionarem melhores resultados do que as técnicas convencionais.

Para o estágio I, quando o LES dorsal está intacto, também conhecida com lesão pré-dinâmica, está indicada fixação percutânea ou assistida por artroscopia, com o sem ligamentosplastia e capsulodese dorsal. 50

Para o estágio II, quando há ruptura do LES dorsal e volar, pode-se realizar ligamentosplastia e capsulodese dorsal 50 e volar, 36 com fixação escafolunar com fios de Kirschner ou parafuso escafolunar (RASL) assistida por artroscopia.

Nos estágios III (lesão completa do LES irreparável) e IV (estágio III com rotação do escafoide redutível), pode-se realizar diversos tipos de reconstrução do ligamento escafolunar. As técnicas clássicas são a capsulodese dorsal ou reconstrução de Brunelli ou Brunelli modificada por Garcia-Elias. As técnicas que vêm ganhando mais adeptos atualmente são as que realizam o reparo 360 ^o^ , como a de Corella et al. 51

Para os estágios V (rotação do escafoide irredutível) e VI (artrose secundária a lesão do LES) estão indicadas as artrodeses parciais e total do punho e a ressecção da fileira proximal dos ossos do carpo. Há na literatura diversos autores que demonstram ser factível realizar estes procedimentos assistidos por artroscopia. Ainda não evidência, contudo, que os procedimentos minimamente invasivos são vantajosos sobre as técnicas tradicionais.

## Luxação Perilunar do Carpo e Fratura-luxação Perilunar do Carpo

Uma vez que a artroscopia do punho pode auxiliar na redução e fixação das fraturas do rádio e do escafoide e no reparo dos ligamentos do carpo, tornou-se uma opção para auxílio na redução e tratamento das fraturas-luxações do carpo. Apesar de haver um trabalho da literatura que demonstre superioridade do auxílio artroscópico, 52 precisaremos de mais artigos para nos certificarmos que esta é a melhor técnica. Contudo, aconselhamos cuidado na indicação da artroscopia nestas lesões, uma vez que a lesão capsular extensa e grande instabilidade trazem grande dificuldade técnica no procedimento artroscópico.

## Lesão do Complexo da Fibrocartilagem Triangular (CFCT)

Esta é uma das indicações mais indiscutíveis para a artroscopia do punho, nos dias de hoje. Desde o clássico artigo de Palmer, 53 que classificou as lesões traumáticas e degenerativas do CFCT, muito se estudou sobre a anatomia e funções deste complexo ligamentar. Quem experimentou a cirurgia aberta para a desinserção ulnar do CFCT muitas vezes esteve em dificuldades tanto em identificar o tecido correto quanto em definir o local de reinserção do CFCT.

Realizar desbridamentos do CFCT e avaliar a condropatia do semilunar, piramidal e cabeça da ulna por via aberta parece uma realidade distante. A lesão tecidual seria muito grande e a avaliação incompleta.

O artigo de 2008 Anderson et al. 54 não demonstrou diferenças significativas na função e mobilidade do paciente entre o reparo artroscópico e o reparo aberto do CFCT. Contudo, apenas o último realizava a reinserção foveal do ligamento. Em 2009, Atzei 1 publicou seu trabalho de reinserção foveal artroscópcia do CFCT que ganhou popularidade e obteve reconhecimento mundo afora. Neste mesmo artigo, ele propôs uma classificação para lesões traumáticas periféricas ulnares do CFCT (Classe 1B de Palmer) ( Fig. 3 ).

**Fig. 3:** Tipos de lesão do complexo da fibrocartilagem triangular. A: desinserção distal do CFCT, classe 1. B: Desinseção proximal e distal do CFCT, classe 2. C: Demonstração do teste do “gancho” positivo para a lesão demonstrada na figura B (classe 2). D: lesão do CFCT classe 4 com bordas friáveis. E: mesma paciente demonstrada na figura B, com grande instabilidade dos remanescentes do CFCT. F: lesão maciça do CFCT, irreparável, classe 4.

Esta classificação permitiu que se traçasse um algoritmo para o tratamento das lesões periféricas ulnares do CFCT e suas sequelas. Lesões sem desinserção foveal podem ser reparadas com pontos periféricos entre a cápsula e o CFCT, lesões com avulsão proximal devem ser reinseridas na fóvea, lesões irreparáveis devem ser reconstruídas e pode-se proceder a artroplastias e artrodeses (Sauvè-Kapandji) para pacientes com lesões cartilaginosas.

Desde 2009, diversos outros autores lançaram técnicas para reinserção foveal do CFCT via artroscópica 55 56 e aberta. 57

Técnicas artroscópicas para reconstrução do CFCT com enxertos de tendão começaram a surgir. A técnica aberta de Adams e Berger 58 é o padrão ouro para a reconstrução do complexo ligamentar. Contudo, há diveras técnicas ganhando popularidade para reconstrução artroscópica. 59 60 61 Acreditamos que, em algum tempo, técnicas anatômicas e com menor lesão articular e de mecanoceptores substituirão as técnicas abertas.

Obviamente, deve-se observar a articulação rádio-ulnar distal com toda sua complexidade e não apenas o CFCT para se tomar decisões quanto ao tratamento a ser implementado. 62

## Discussão

A artroscopia nas articulações do punho e da mão tem-se desenvolvido muito rapidamente nos últimos anos. A Sociedade Europeia de artroscopia do punho é uma das maiores responsáveis por este salto de conhecimento e prática, uma vez que uniu diferentes escolas da Europa e do Mundo em torno do tema.

Para diversos procedimentos, acreditamos estarmos melhores com o auxílio da artroscopia do que sem ele. Contudo, devemos lembrar que a artroscopia é um método, uma ferramenta para chegar aonde queremos e não o propósito do tratamento.

## Conclusão

A artroscopia do punho e da mão nos permite auxiliar no diagnóstico e tratamento da imensa maioria das lesões e doenças que acometem a região.
